# 273. Chlorhexidine Gluconate (CHG) Bathing in Nursing Homes (NHs) and Hospitals: Impact on the Nose & Skin Microbiome and Multidrug Resistant Organism (MDRO) Prevalence

**DOI:** 10.1093/ofid/ofad500.345

**Published:** 2023-11-27

**Authors:** Gabrielle Gussin, Sean Conlan, Raveena D Singh, Clay Deming, Raheeb Saavedra, Connie Nguyen, Thomas T Tjoa, Robert Pedroza, Chase Berman, Julie A Shimabukuro, Pamela B Bell, Sangeetha Baskaran, Mary Stanley, Mary K Hayden, Cassiana E Bittencourt, Julie Segre, Susan S Huang

**Affiliations:** University of California, Irvine School of Medicine, Division of Infectious Diseases, Irvine, California; National Institutes of Health, Bethesda, Maryland; University of California, Irvine School of Medicine, Division of Infectious Diseases, Irvine, California; National Institutes of Health, Bethesda, Maryland; University of California, Irvine School of Medicine, Division of Infectious Diseases, Irvine, California; University of California, Irvine, Irvine, California; University of California, Irvine School of Medicine, Division of Infectious Diseases, Irvine, California; University of California, Irvine School of Medicine, Division of Infectious Diseases, Irvine, California; University of California, Irvine School of Medicine, Division of Infectious Diseases, Irvine, California; University of California, Irvine Health, Orange, California; Rush University Medical Center, Chicago, Illinois; Rush University Medical Center, Chicago, Illinois; Rush University Medical Center, Chicago, Illinois; Rush University Medical Center, Chicago, Illinois; University of California, Irvine Health, Orange, California; National Institutes of Health, Bethesda, Maryland; University of California, Irvine, Irvine, California

## Abstract

**Background:**

Widespread CHG bathing to prevent infection has raised concerns about potential skin microbiome perturbations and depletion of commensal microbiota.

**Methods:**

A prospective repeated measures cross-over study in NH residents/hospital patients evaluated the impact of CHG vs routine soap bathing on the nose/skin microbiome. Participants underwent serial visits during distinct CHG and routine soap phases (Figure) involving nares, axilla, groin, and finger/hand swabs processed for MDROs, CHG concentration (skin sites only), and bacterial 16S rRNA V1-3 gene sequencing (Illumina MiSeq). Sequences were processed with DADA2 and analyzed with PhyloSeq and Vegan packages in R to calculate microbial diversity and composition.

**Results:**

We enrolled 30 participants (20 NH, 10 hospital). Mean age was 62y, 52% female. Mean CHG concentration was 1736 µg/mL (range: 0-20000) during CHG visits vs 17.5 µg/mL (range: 0-1250) during routine soap visits. MDRO prevalence was lower during CHG visits (NH: 19%, hospital: 9%) vs routine soap visits (NH: 47%, hospital: 23%). 555 samples from 30 participants were adequate for microbiome analyses.

CHG did not affect overall alpha diversity of microbial communities on skin (Figure), but proteobacteria were notably higher in NH vs hospital participants (P< 0.001). Because gram-negative bacteria often exhibit higher MICs to CHG than gram-positive bacteria, we assessed whether CHG concentration in NHs was associated with greater relative abundance of proteobacteria using generalized linear mixed models clustered by person. In NHs, CHG concentration was not associated with relative abundance of skin proteobacteria, although body mass index ≥30 (14% higher abundance, P=0.01) and stool/urine incontinence were (13% higher, P=0.01). Compared to the axilla, the groin had 35% higher relative abundance of proteobacteria (P< 0.001); fingers/hand samples had 9% lower (P=0.01).

Nose/Skin Taxonomic Diversity Not Impacted by CHG Bathing

**Figure:**
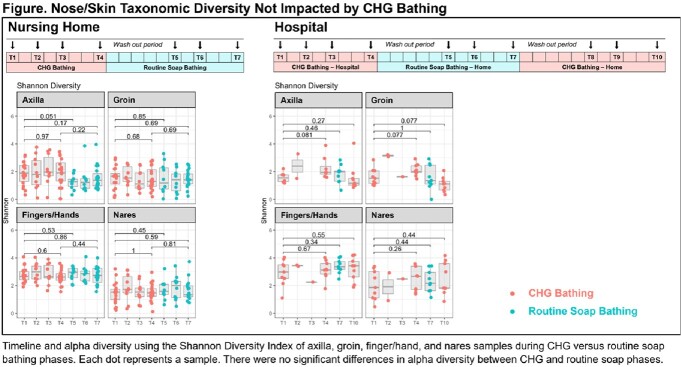

Timeline and alpha diversity using the Shannon Diversity Index of axilla, groin, finger/hand, and nares samples during CHG versus routine soap bathing phases. Each dot represents a sample. There were no significant differences in alpha diversity between CHG and routine soap phases.

**Figure:**
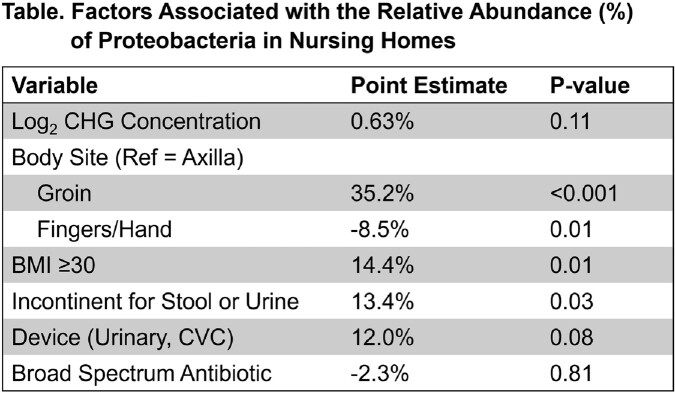

**Conclusion:**

CHG bathing reduced MDRO prevalence but did not impact skin or nasal microbial alpha diversity in hospital or NH participants. Overall, NH residents had notably higher relative abundance of skin proteobacteria vs hospital patients. This finding was not associated with CHG concentration. Rather, proteobacteria appeared enriched in NH residents with obesity or incontinence.

**Disclosures:**

**Gabrielle Gussin, MS**, Medline Industries, Inc: Conducted studies where participating hospitals/nursing homes received cleaning & antiseptic product|Xttrium Laboratories: Conducted studies where participating hospitals & nursing homes received antiseptic bathing product **Raveena D. Singh, MA**, Medline Industries, Inc: Conducted studies where participating hospitals/nursing homes received cleaning & antiseptic product|Xttrium Laboratories: Conducted studies where participating hospitals & nursing homes received antiseptic bathing product **Raheeb Saavedra, AS**, Medline Industries, Inc: Conducted studies where participating hospitals/nursing homes received cleaning & antiseptic product|Xttrium Laboratories: Conducted studies where participating hospitals & nursing homes received antiseptic bathing product **Connie Nguyen, n/a**, Xttrium Laboratories: Conducted studies where participating hospitals & nursing homes received antiseptic bathing product **Robert Pedroza, BS**, Medline Industries, Inc: Conducted studies where participating hospitals/nursing homes received cleaning & antiseptic product **Chase Berman, BS**, Medline Industries, Inc: Conducted studies where participating hospitals/nursing homes received cleaning & antiseptic product **Susan S. Huang, MD MPH**, Medline Industries, Inc: Conducted studies whereby participating nursing homes and hospital patients received cleaning & antiseptic products|Xttrium Laboratories: Conducted studies where participating nursing homes and hospital patients received antiseptic products

